# Histomorphological responses after therapy with pegylated interferon α-2a in patients with essential thrombocythemia (ET) and polycythemia vera (PV)

**DOI:** 10.1186/s40164-017-0090-5

**Published:** 2017-11-09

**Authors:** Lucia Masarova, C. Cameron Yin, Jorge E. Cortes, Marina Konopleva, Gautam Borthakur, Kate J. Newberry, Hagop M. Kantarjian, Carlos E. Bueso-Ramos, Srdan Verstovsek

**Affiliations:** 1 Department of Leukemia, The University of Texas MD Anderson Cancer Center, 1515 Holcombe Blvd., Unit 428, Houston, TX 77030 USA; 2 Department of Hematopathology, The University of Texas MD Anderson Cancer Center, Houston, TX 77030 USA

**Keywords:** Essential thrombocythemia, Polycythemia vera, Pegylated interferon alfa-2a, Histomorphological response

## Abstract

**Background:**

Pegylated interferon alfa-2a (PEG-IFN-α-2a) is a potent immunomodulating agent capable of inducing high rate of hematologic and even complete molecular remission in patients with essential thrombocythemia (ET) and polycythemia vera (PV). We recently reported results of a phase 2 trial of PEG-IFN-α-2a in 83 patients with ET and PV after a median follow-up of 83 months. Here we report an analysis of bone marrow (BM) responses in these patients.

**Methods:**

Among 83 patients, 58 (70%, PV 25, ET 31) had evaluable BM samples. BM responses and fibrosis grading were defined according to the International Working Group for Myeloproliferative Neoplasms Research and Treatment, and the European Consensus on grading of BM fibrosis, respectively. BM was assessed prior to enrollment, and every 6–24 months while on therapy in all patients, and after therapy discontinuation in some patients.

**Results:**

The median age of analyzed 58 patients was 52 years, and 29% were males. After a median follow-up of 84 months, 32 patients are still on study. Hematologic (HR) and molecular responses (MR) were seen in 93 and 69% patients, respectively. Twenty-nine patients (50%) had a BM response, including 13 (22%) with a complete BM response (BM-CR). Moreover, 13 patients (22%) have experienced complete resolution of bone marrow reticulin fibrosis. Patients with BM response had higher duration of HR and MR, and lower discontinuation rate. Furthermore, patients with BM-CR had a higher probability of complete MR. The median duration of BM-CR was 30 months, and 9 patients have maintained their BM-CR (69%), including five who have maintained their response after discontinuation of therapy. Despite this observation, the pattern of HR, MR and BM response, their durability and interrelation was heterogeneous.

**Conclusions:**

Our results show the ability of PEG-IFN-α-2a to induce complete BM responses in a subset of ET and PV patients, but its correlation with durable clinically relevant treatment benefit warrants further investigation.

*Trial registration* This study is registered with ClinicalTrials.gov (NCT00452023), and is ongoing but not enrolling new patients.

## Background

Essential thrombocythemia (ET) and polycythemia vera (PV) are BCR-ABL–negative myeloproliferative neoplasms (MPN) characterized by an overproduction of mature blood elements, tendencies toward thrombosis and hemorrhage, extramedullary hematopoiesis (mild splenomegaly), and transformation to overt myelofibrosis (MF)/acute myeloid leukemia (AML) [1, 2]. The therapeutic approach for patients at high risk of thrombosis include cytoreductive therapy such as hydroxyurea [3] or recombinant interferon-alfa [4–6]. Several clinical studies have reported that pegylated interferon-alfa can induce complete hematologic remission (CHR) in majority of patients with ET and PV, and is even capable to induce complete molecular remission (CMR) in some patients [6–8]. Similarly, clinical benefit and high HR were noticed in patients with early stages of MF, where interferon-alfa has been shown to possibly reverse bone marrow (BM) fibrosis [9, 10].

Recently, we reported a long-term follow-up of a prospective, phase 2 clinical trial of pegylated interferon alfa-2a (PEG-IFN-α-2a) in 83 patients with PV or ET. After a median follow-up of 83 months, overall HR and MR were noticed in 80 and 63%, respectively, and were shown durable in some patients, with overall median response durations of 66 and 53 months, respectively [8]. Our findings confirmed observations by others that neither baseline molecular status nor achievement of a MR on therapy, are a prerequisite for achieving a HR, and some patients continued to derive significant clinical benefit despite losing their HR or best MR. Histomorphologic evaluation of BM is an important part of establishing a diagnosis [11] and in response assessment in patients with ET/PV [12]. According to the most recent WHO classification, a major criterion for diagnosis of ET is a BM biopsy showing proliferation of the megakaryocyte lineage, with no significant increase in granulopoiesis or erythropoiesis, and very rarely minor (grade 1) increase in reticulin fibers; and for PV, a BM biopsy showing hypercellularity with panmyelosis [11]. The development of BM fibrosis is considered part of the natural evolution of these diseases, and has been reported in up to 20 and 51% of patients with ET and PV during their disease duration, respectively [13–15]. Although there is an increasing evidence of direct correlation between a higher BM fibrosis grade in patients with ET/PV and signs of progressive disease, such as splenomegaly, cytogenetic aberrations, increased risks of vascular complications, and transformation to overt MF [14–17], the prognostic value of improvement in BM fibrosis (or other BM characteristics) in patients with PV and ET while on therapy, remains unknown. Despite promising preclinical data showing that the robust immunomodulatory and anti-proliferative effects of interferon-alfa may reverse BM fibrosis via its stimulatory effects on hematopoietic stem cells and inhibitory effects on megakaryopoiesis [18–20], morphologic remission with interferon-alfa therapy has been rarely reported [21–23]. Herein, we report the histomorphological BM responses of patients with ET/PV treated with PEG-IFN-α-2a in a prospective, open-label, single-center, phase 2 clinical trial.

## Patients and methods

Overall, 83 patients (43 PV, 40 ET) were enrolled in a clinical trial of PEG-IFN-α-2a between 2005 and 2009 at our institution. Detailed inclusion and exclusion criteria, treatment schedule, response assessment, statistical considerations and primary analyses have been previously reported [6, 24].

Hematologic and molecular responses, as defined by the European LeukemiaNet [12], were assessed every 3 and 6 months, respectively. Histomorphology of the BM, including fibrosis grading, and morphological responses according to European LeukemiaNet, International Working Group-Myeloproliferative Neoplasms Research and Treatment (IWG-MRT) and European Consensus [12, 25] were assessed by expert hematopathologists at our institution before study enrollment, every 6–12 months during the first 3 years while on study, and then every 12–24 months during the subsequent years. Some patients continued to have BM evaluated every 12–24 months after treatment discontinuation. Complete BM remission (BM-CR) was defined by ELN and IWG-MRT criteria [12] as follows: absence of more than grade 1 reticulin fibrosis and disappearance of megakaryocyte hyperplasia in ET or trilinear hyperplasia with age-adjusted normocellularity in PV. An incomplete, partial response (BM-PR) was defined as fibrosis grading that had improved by at least one grade level on at least 2 consecutive samples taken ≥ 12 months apart, but with persistent morphological features of ET or PV.

Responses and clinical data were analyzed using descriptive statistics. Fisher’s exact and Wilcoxon rank-sum tests were used to compare categorical and continuous variables, respectively. Time to BM-responses and its duration was estimated by using Kaplan–Meier method. GraphPad Prism and SPSS v.23 were used for all analyses.

## Results

Among 83 patients enrolled to this study, 58 (70%) had BM samples that were evaluable for BM response assessment. Eighteen patients treated for ≤ 12 months and 7 without consecutive BM samples were not evaluable for BM response. Demographics and clinical characteristics of 58 currently analyzed patients are detailed in Table 1. The median follow-up on study was 84 months (range 36–107), and median number of BM samples per patient was 8 (range 3–12). Median age was 52 years (range 19–75) and 29% (n = 17) were men. Sixty-two percent of patients (n = 36) were previously treated, the majority of them with hydroxyurea (n = 27, 75%). Except for abnormalities in marrow cellularity, atypical megakaryocytes characteristic of ET/PV [11], 35 patients (25 PV, 10 ET; 60%) had reticulin fibrosis of grade MF-1 or MF-2 according to European grading [25]. Median exposure to PEG-IFN-α-2a was 80 months (range 15–107). The starting doses of PEG-IFN-α-2a given subcutaneously in a weekly schedule were 450 µg in 2 patients, 360 µg in 2 patients, 270 µg in 11 patients, 180 µg in 20 patients and 90 µg in 35 patients. The dose was modified throughout the study based on toxicity or lack of efficacy. At the time of this report, out of 58 patients, 32 (55%) are still on study, 24 (75%) of whom are actively being treated.

**Table 1:** Clinical and demographic characteristics of all patients, and stratified by BM response

Characteristics	All pts, n = 58	BM-NR, n = 29	BM-PR, n = 16	BM-CR, n = 13
PV/ET, no.	27/31	15/14	5/11	7/6
Median age, years (range)	52 (19–75)	53 (19–72)	55 (33–70)	52 (23–75)
JAK2 V617F-positive patients, no (%)	42 (72)	22 (76)	9 (56)	11 (84)
Other driver mutations, no (%)	CALR 6, MPL 2, TN 8	CALR 1, TN 6	CALR 5, MPL 2	TN 2
JAK2 V617F allele burden, median (range)	34 (3–89)	32.4 (3–82)	41 (18–89)	30 (5–76)
Abnormal karyotype, no	3	–	2	1
Median white blood cell count, 109/L (range)	9 (4–19)	9 (4–19)	9 (3–19)	8 (4–12)
Median hemoglobin, g/dL (range)	13 (9–19)	14 (12–17)	13 (9–18)	13 (12–19)
Median platelet count, 10^9^/L (range)	721 (128–1646)	629 (185–1646)	866 (128–1423)	420 (236–1020)*
Significant splenomegaly, n (% of known)^a^	9/55 (16)	5/28 (18)	3/15 (20)	1/12 (8)
Disease-related symptoms, n (%)	30 (52)	17 (59)	6 (38)	7 (54)
No. of previously treated (%)	36 (62)	16 (55)	11 (69)	9 (69)

* p value < 0.05

^a^Significant splenomegaly defined as a palpable spleen > 5 cm below costal margin (BCM), Abnormal karyotype: BM-PR: DER (13;22)(Q10;Q10) (n = 1), + MAR (n = 1), BM-CR: +1, DER (1;22)(Q10;Q10) (n = 1)

### Hematologic response

Initial HR was achieved in 54 (93%) patients, and it was sustained in 33% of them (n = 18) throughout the entire follow-up on a study with a median response duration of 92 months (range 58–101 +). The remaining 36 patients lost their HR after a median duration of 55 months (range 10.4–91). Sixteen patients who lost their HR, are still on study, 14 of them actively treated, as they continue to derive clinical benefit (no clinical symptoms and organomegaly) with good tolerance.

### Molecular response

Among 44 patients who were JAK2V617F-positive, the MR rate was 72% (n = 32; a complete MR [CMR; undetectable JAK2V617F allele burden] in 9, a partial MR [PMR; 50% reduction in JAK2V617F allele burden] in 19, and a minor MR [mMR; 20–49% reduction in JAK2V617F allele burden] in 4 patients, Table 2). The median JAK2V617F allele burden was 25% at baseline for those achieving CMR, while it decreased from 41 to 8% in those with PMR, and 34–24% in those with mMR. Pre-treatment allele burden did not differ among patients with CMR, PMR and mMR. The response duration was similar in patients with CMR (68 months; range 21–94), PMR (53 months; range 7–79) and mMR (50 months; range 12–60).

**Table 2:** Responses details, stratification by BM response

Characteristics	All patients, n = 58	BM-NR, n = 29	BM-PR, n = 16	BM-CR, n = 13
Initial HR, no. (%)	54 (93)	26 (90)	15 (94)	13 (100)
Last HR, no (% of initial HR)	22 (41)	7 (27)	5 (33)	9 (69)
Duration of HR, months (range)	90 (10–101)	50 (10–98)	69 (17–96)	87 (59–101)*
Initial MR, no. (% of JAK2+), type	33/44 (75) 9 CMR, 20 PMR, 4 mMR	15 (68) 2 CMR, 11 PMR, 2 mMR	8 (89) 6 PMR, 2 mMR	10 (91) 7 CMR*, 3 PMR
Last MR, no (% of initial overall MR), type	22 (67) 9 CMR; 4 PMR, 9 mMR	7 (47)^a^ 2 CMR, 5 mMR	6 (75) 3 PMR, 3 mMR	9 (90) 7 CMR, 1 PMR, 1 mMR
Time to BM response, months (range)	52 (12–72)	NA	54 (12–72)	52 (30–72)^b^
BM response duration, months (range)	36 (12–66)	NA	36 (12–66)	30 (24–52)
Last BM response, no (% of initial), type	9 BM-CR; 14 BM-PR	NA	14 BM-PR (88) 2 BM-NR (12)	9 BM-CR (69) 4 BM-NR (31)
Vascular adverse event on therapy, No (%)	6 (10)	3 (10)	2 (13)	1 (8)
MF transformation on therapy, no (%)	4 (7)	4 (14)	0	0
Duration of therapy, months (range)	80 (15–107)	75 (25–205)	85 (77–107)	83 (15–101)
Total median follow-up, months (range)	84 (36–107)	81 (36–105)	94 (67–107)	93 (83–106)
On study at last follow-up, no (%)	32 (55)	10 (35)	13 (81)	9 (69)*

*DX* diagnosis, *HR* hematologic response, *MR* molecular response

* p < 0.05

^a^One patient was not evaluable for subsequent MR

^b^8/13 patients with BM-CR (61%) achieved complete resolution of reticulin fibrosis (MF-0) after a median of 63 months on therapy (range 30–72); the remaining 5 patients has decreased their BM fibrosis to MF 0/1 after a median of 48 months (range 30–69) on therapy

All patients with CMR (100%), 4 patients with PMR (21%) and 2 with mMR (50%) have maintained their best MR throughout the entire follow-up. Overall 14 patients with PMR (74%) lost their PMR (7 completely and 7 has maintained a mMR). One patient with a PMR received an allogeneic stem cell transplantation for concurrent large B cell lymphoma and was therefore not evaluable. The median JAK2 allele burden at baseline and at the time of best response did not differ between those who maintained a PMR and those who did not. All but one patients were actively treated at the time of their PMR loss (drug was withheld in one patient for 20 months due to G1 adverse events when he lost his PMR).

### Bone marrow response

**Fig. 1:**
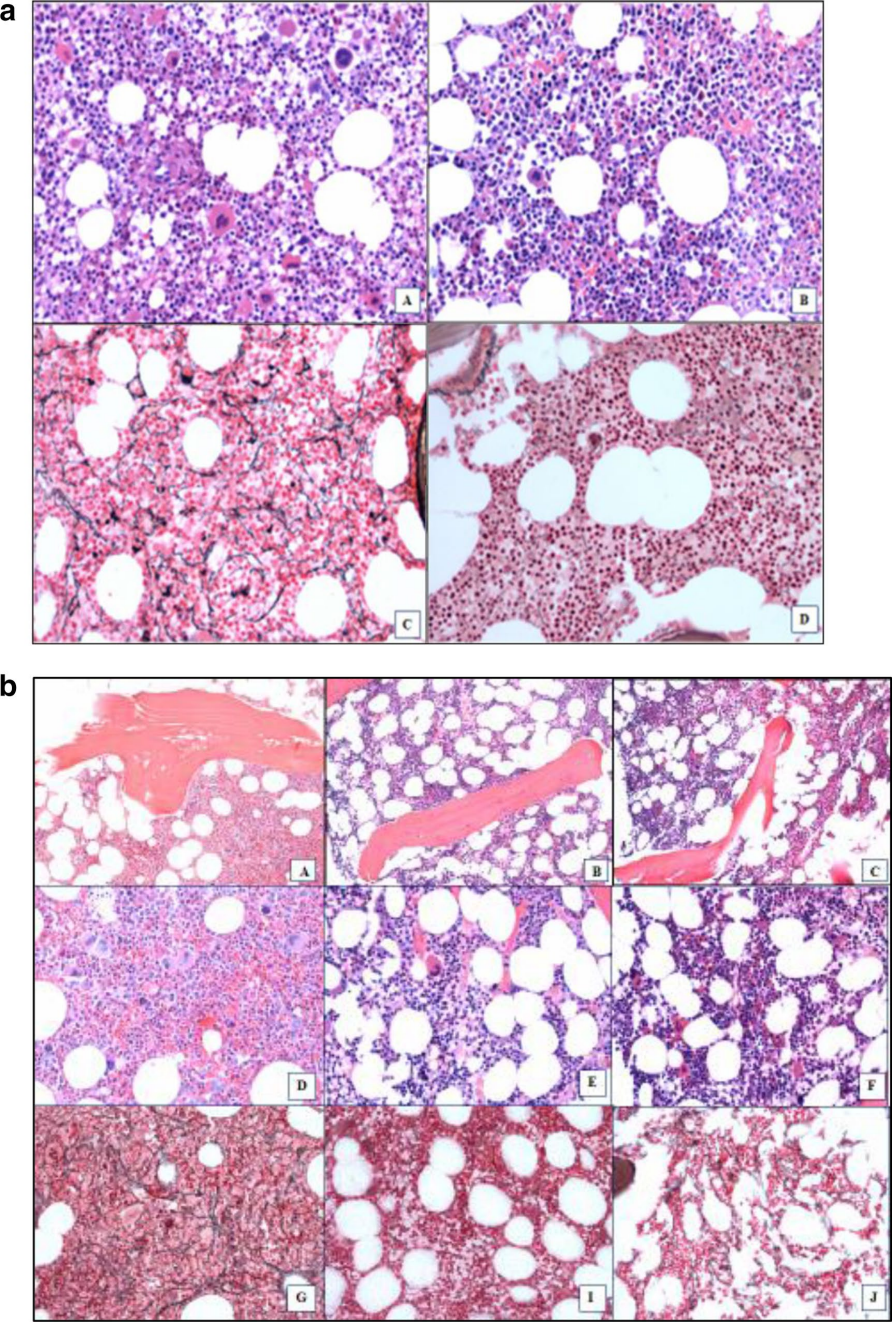
BM Response patterns observed in patients treated with PEG-INF-α-2a. **a** BM biopsy sections from a patient with ET who is still on therapy after 84 + months with BM-CR and CMR that was achieved after 60 months on treatment (Pt 8, Fig. 3a). **A**, **B** Hematoxylin and eosin staining at (**A**) the start of therapy and (**B**) at BM-CR after 60 months on therapy. **C**, **D** reticulin staining showing BM fibrosis at (**C**) the start of therapy (MF-1/2) and **D** at the time of BM-CR (MF-0). **b** Patient with PV who is still on therapy after 96 + months who achieved a CHR, CMR and BM-CR but has only sustained a CMR (Pt 4, Fig. 3b). Loss of BM-CR occurred after 84 months and loss of CHR after 96 months. Hematoxylin and eosin staining showing osteosclerosis at (**A**) the start of therapy, **B** at the time of best response (60 months), and **C** at relapse after 84 months. **D**–**F** Hematoxylin and eosin staining showing BM cellularity and megakaryocyte morphology at (**D**) the start of therapy, **E** at the time of best response (60 months), and **F** at relapse (84 months). **G**, **I**, **J** Reticulin staining showing BM fibrosis at (**G**) the start of therapy (MF-2), **I** at the time of best response (MF-0), and **J** at relapse (MF-1). All images shown are magnified ×200

**Fig. 2:**
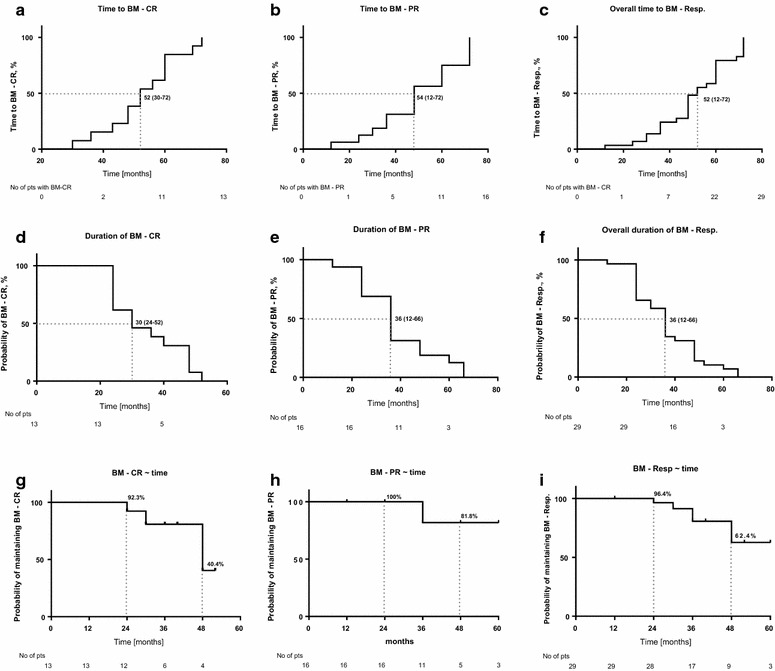
**a**–**i** Kaplan–Meier curves with estimation of time to BM response, its durability and probability of maintenance over time. **a**–**c** Time to BM-CR, BM-PR and overall BM-response; **d**–**f** median duration of BM-CR, BM-PR and overall BM-response; **g**–**i** probability of maintaining achieved BM-CR, BM-PR and overall BM-response over time

Overall, 29 patients (50%) had BM response, including 13 patients (22%) with BM-CR (Table 3, Fig. 1), and 16 patients (28%) with BM-PR [12]. Furthermore, 13 patients (22%) had complete resolution of bone marrow reticulin fibrosis (MF-0), including 3 patients with initial grade 2. Demographic and clinical characteristics and MR patterns in all evaluable patients (n = 58) stratified by BM response (BM-CR, BM-PR, and BM-NR) are shown in Tables 1 and 2. Except for increased platelets at baseline in those with BM-PR (p < 0.001), likely due to the higher proportion of ET patients in that group, no other differences in basic demographic or clinical parameters were present among different response groups (Table 1). There was no difference in the median time to achievement of a best BM response or its duration among patients with BM-CR and BM-PR (Table 2, Fig. 2a–f). Nine (69%) patients with BM-CR and 14 (88%) patients with BM-PR have sustained their best BM response.

**Table 3:** Characteristics of patients with BM-CR

Pt	Pts	DG	DX_ time to enroll., mos	HR DUR, mos	Mol. status	DUR of therapy, mos	Initial BM^b^	Best BM resp.—time from enroll., mos	Last BM	Resp. DUR, mos
1^a^	59 M	PV	97	79.7*	PMR	15.3	MF 2, 90%, OST	MF 0, NEG morph. and OST, 40%—43	MF 1, NEG morph., 60%	30
2^a^	75 F	PV	119	89.4	CMR	91.4	MF 2, 80%	MF 0, NEG morph., 50%—48	MF 1, NEG morph., 50%	48
3^a^	38 M	PV	1	100.9	CMR	100.9	MF 2, 90%, OST	MF 1, NEG morph. and OST, 30%—56	MF 1, NEG morph and OST., 50%	40
4	58 M	PV	35	87.1*	CMR	99.0	MF 2, 90%, OST	MF 0, NEG morph. and OST, 30%—69	MF 1, POS morph., 60%	48
5	37 F	PV	5	94.8	CMR	94.8	MF 1, 70%, OST	MF 0, NEG morph., 40%—60	MF 1, NEG morph., 40%, + OST	48
6^a^	44 M	PV	3	74.7*	CMR	98.3	MF 1, 70%	MF 0, NEG morph., 30%—48	MF 1, NEG morph., 50%	36
7	46 M	PV	350	79.5	PMR	79.5	MF 1, 60%	MF 0, NEG morph., 40%—48	MF 1, POS morph., 50%	30
8^a^	47 F	ET	33	83.0	CMR	83.0	MF 1, 50%	MF 1, NEG morph., 20%—60	MF 0, NEG morph., 30%	24
9^a^	35 M	ET	14	79.8	CMR	30.4	MF 1, 50%	MF 0, NEG morph., 30%—30	MF 0, NEG morph., 50%	52
10^a^	53 F	ET	43	91.7	NR	58.7	MF 1, 50%	MF 0, NEG morph., 40%—48	MF 0, NEG morph., 20%	24
11^a^	39 F	ET	2	92.6	TN	18.1	MF 1, 50%	MF 0, NEG morph., 40%—66	MF 0, NEG morph., 40%	24
12^a^	23 F	ET	30	100.8	TN	91.5	MF 1, 20%	MF 1, NEG morph., 30%—60	MF 0, NEG morph., 30%	24
13	73 F	ET	110	59.3*	PMR	30.4	MF 2, 60%, OST	MF 1, NEG morph., 30%—30	MF 1, POS morph., 40%	24

*DUR* duration, *DX* diagnosis, *OST* osteosclerosis, *Resp* response, *morph.* morphology, *enroll*. enrollment to study, *NEG* negative, *POS* positive

^a^Patients who sustained a BM response at last follow-up, * lost HR

^b^BM description includes degree of reticulin fibrosis on scale of 0–3, % BM cellularity, and presence of osteosclerosis (OST)

The median initial dose of PEG-IFN-α-2a was 180 µg per week (range 90–450 mcg) in each group. All patients required dose adjustments either for toxicity (n = 44) or for lack of efficacy (n = 14). Median cumulative weekly dose of PEG-IFN-α-2a until best BM response was 45 µg per week, similar for patients with BM-CR and BM-PR. No differences in median weekly dose of PEG-IFN-α-2a while on therapy or overall treatment duration were found among patients with BM-CR, BM-PR and BM-NR.

Interestingly, although the overall duration of therapy was similar among groups (Fig. 2d–f), significantly fewer patients with BM-NR remained on the therapy at the time of last follow-up than those with BM-CR or BM-PR (35% vs 69% vs 81%, p = 0.03). Patients with a BM-CR had a significantly longer HR duration than patients with BM-PR and BM-NR (87 vs 69 vs 50 months; p = 0.01), respectively. However, no difference in rate of the initial HR was seen among groups.

Patients with a BM response (BM-CR & BM-PR) had a higher initial MR rate than patients with BM-NR (91 and 89% vs 50%, p = 0.04). Furthermore, patients with a BM-CR had higher CMR rate than all other patients (BM-PR & BM-NR) (70% vs 12%; p < 0.001; Table 2). Similarly, patients with PV who have achieved CMR have experienced significant reduction in bone marrow cellularity (80–40%, p = 0.02) when compared to the remaining PV patients. On the other hand, durability of MR was the same regardless of BM response status. Despite the lower MR rate in patients with BM-NR, we have still observed CMR and PMR in 2 and 11 of these patients, respectively.

**Fig. 3:**
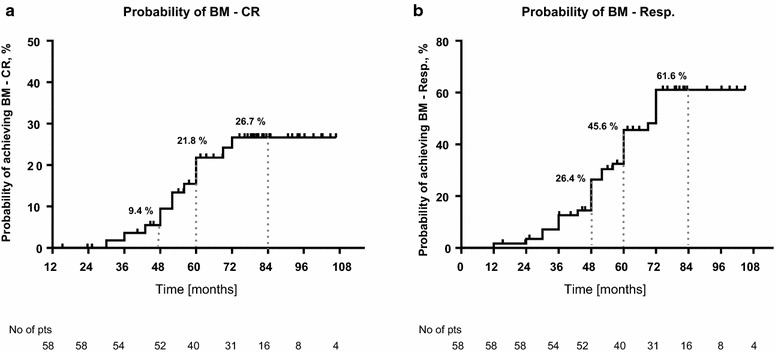
Probability of achieving a BM-response in patients treated with PEG-IFN-α-2a estimated by Kaplan–Meier curve. **a** Probability of achieving BM-CR; **b** Probability of achieving overall BM-response (BM-CR and BM-PR)

Probability of maintaining achieved BM response was 92 and 40% at 2 and 4 years for BM-CR, and 96 and 62% for all BM-responses, respectively (Fig. 2g–i). Probability of achieving any BM response and BM-CR on a study in all patients treated with PEG-INF-α-2a was 26 and 9% at 4 years, and 61 and 27% at 7 years, respectively (Fig. 3a, b).

**Fig. 4:**
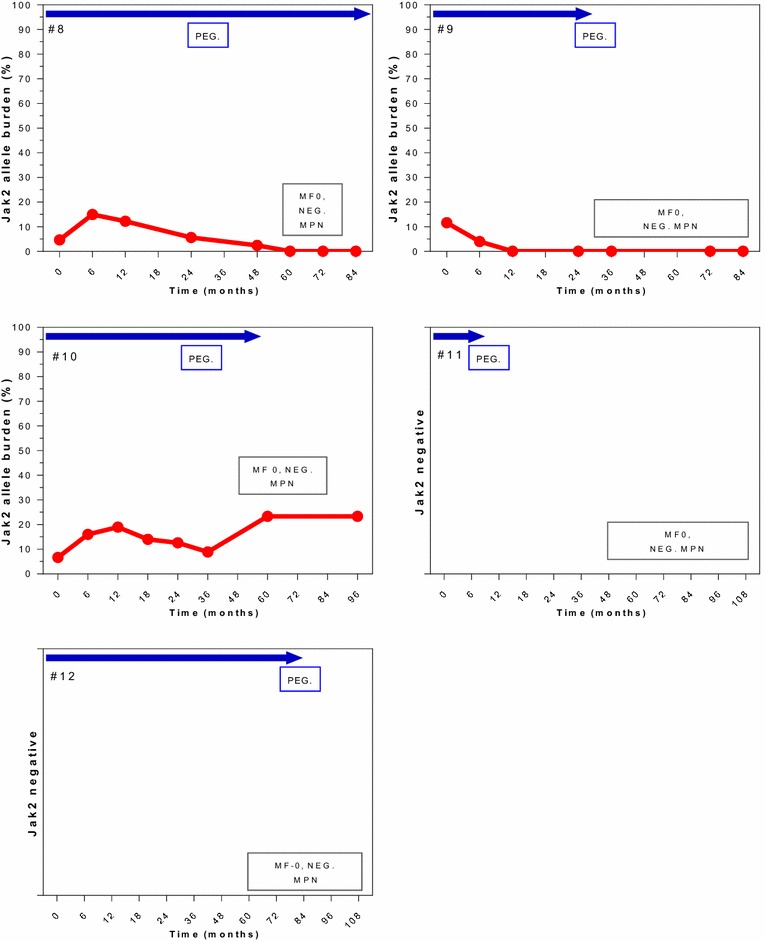
Correlation of molecular and morphological responses over time in selected patients. Patients with a sustained BM-CR (MF-0). Blue arrows indicate time on active therapy. The number in the top left upper corner represents the patient # from Table 2. The red line indicates the JAK2 allele burden over time (missing in TN patients). The box in the bottom right corner shows the change in BM and response, NEG MPN = no signs of ET or PV, normal BM. The gray square represents the time of loss of HR (shown only in patients who lost their HR)

**Fig. 5:**
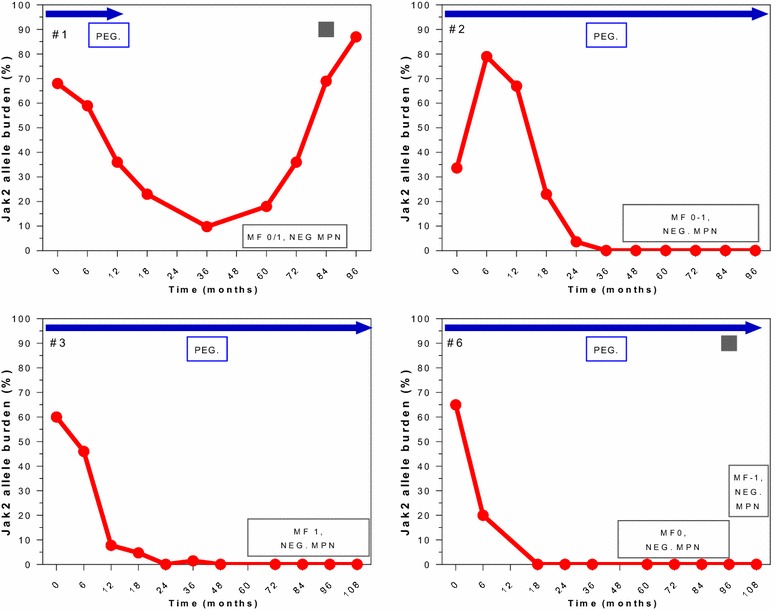
Correlation of molecular and morphological responses over time in selected patients. Patients with a sustained BM-CR with mild reticulin fibrosis (MF 0/1). The number in the top left upper corner represents the patient # from Table 2. The red line indicates the JAK2 allele burden over time. The box in the bottom right corner shows the change in BM and response, NEG MPN = no signs of ET or PV, normal BM. The gray square represents the time of loss of HR (shown only in patients who lost their HR)

**Fig. 6:**
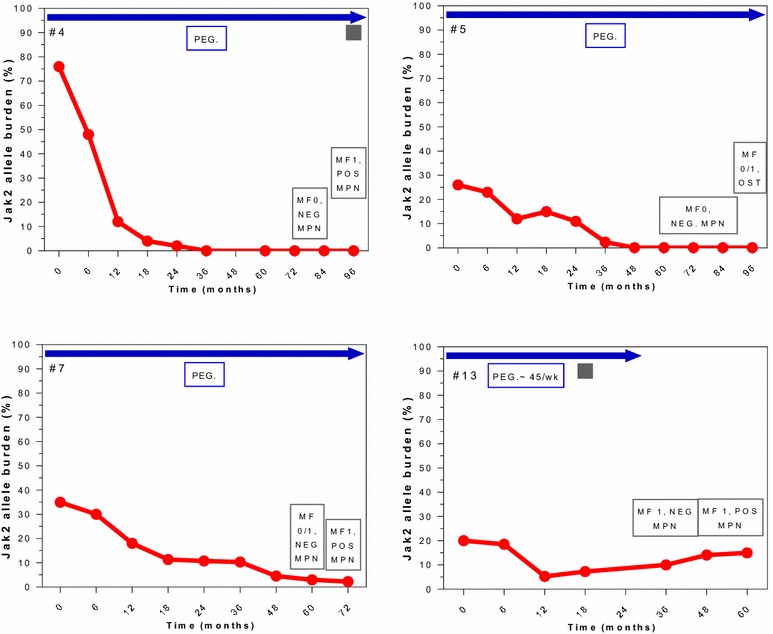
Correlation of molecular and morphological responses over time in selected patients. Patients with BM-CR (MF-0/1, MF-1) and subsequent relapse. Blue arrows indicate time on active therapy. The number in the top left upper corner represents the patient # from Table 2. The red line indicates the JAK2 allele burden over time. The box in the bottom right corner shows the change in BM and response, NEG MPN = no signs of ET or PV, normal BM. The gray square represents the time of loss of HR (shown only in patients who lost their HR)

Correlation between HR, MR and BM response in patients with BM-CR are individually detailed in Figs. 4, 5 and 6. All 13 patients with BM-CR have initially achieved HR, which was sustained in 9 patients at the time of last follow-up. Two of those 4 patients who has lost their HR, remained in sustained BM-CR. Seven and 3 of these patients achieved CMR and PMR, respectively. None of the patients with CMR has experienced a molecular relapse during the entire follow-up, whilst it was observed in 2 of 3 patients with PMR. One patient who was actively treated on a study for 59 months, did not achieve any MR despite achieving a CHR and BM-CR, both currently ongoing despite > 40 months off therapy.

Four patients have lost their BM-CR (Fig. 6) after a median response duration of 39 months (range 30–48), and 3 of them were still on active therapy before they lost their BM-CR. Loss of BM response was accompanied by loss of CHR and PMR in 2 and 1 patient, respectively.

Given the fact that achievements of BM-CR allows for grade 1 fibrosis in the BM, we have also evaluated the ability of PEG-IFN-α-2a to revert BM reticulin fibrosis. Overall, 13 patients (22%, BM-CR in 11, BM-PR in 2) have achieved complete resolution of initially present BM fibrosis (MF-2 or MF-1 to MF-0 in 3 and 10 patients, respectively), which was sustained in 5 patients at the time of last follow-up (all in BM-CR). Additional 18 patients (31%, BM-CR in 2, BM-PR in 16) have experienced decrease in BM fibrosis by 1 grade (MF-2 to MF-1), which was sustained in 15 patients at the time of last follow-up (1 in BM-CR and 14 in BM-PR).

### Vascular complications

Overall, 6 (10%) patients experienced unprovoked vascular complications (5 thrombotic and one bleeding event): one while having BM-CR, 2 BM-PR, and 3 BM-NR. The incidence rate of major unprovoked thromboembolic event in enrolled patients during the entire study follow-up was 1.37 per 100 person years. One patient with BM-CR experienced pulmonary embolism after being treated for 14 months, while being in CHR for past 6 months. The patient continued on therapy with PEG-IFN-α-2a after the event for another 80 months, was able to achieve CMR (JAK2 allele burden at the time of embolism was 75%), and BM-CR, which were all sustained at the time of last follow-up, when the patient discontinued treatment due to financial reasons. Both patients with BM-PR developed vascular complications (femoral arterial thrombosis and hemorrhagic stroke in one each) after therapy for 24 and 36 months, respectively; one patient was in CHR and the other one has never achieved HR. Both continued on therapy after the vascular event occurred for additional 42 and 60 months, respectively, and discontinued the treatment for financial reasons.

### Disease progression

Progression to overt MF and AML occurred in 5 patients (9%; 4 MF and 1 AML), and none of these patients had a BM response. One patient who progressed to AML was holding PEG-IFN-α-2a for 6 months before transformation for unrelated reason (knee replacement surgery). None of the 4 patients were in CHR at the time of progression to MF, and all have discontinued the therapy afterwards.

### Patients after therapy discontinuation

Among patients with a BM response who discontinued therapy, 6 continued to have BM biopsies performed, with a median of 4 samples per patient (range 3–6). Four stopped therapy while in BM-CR and 2 while in BM-PR.

Two patients (#1 and #11, Figs. 4, 5), who had achieved a BM-PR at the time of treatment discontinuation, continued to show improvements in their BM despite being off therapy. Remarkably, both achieved a BM-CR after being off therapy for 32 and 36 months, and maintained their BM-CR for 30 and 24 months, still off therapy, respectively. One of these patients (#1, Fig. 5) has lost his CHR and best MR while still remaining in BM-CR; the other one continues to be in CHR (MR not evaluable for JAK2 negativity). Among those 4 patients who stopped therapy at the time of BM-CR (#9, #10, #12, and #13, Fig. 4), 3 remain in sustained BM-CR. One of them has lost his best MR (#10, Fig. 4), one remains in CMR (#9, Fig. 4) and one is JAK2 negative (#12, Fig. 4). All three patients maintain their initial CHR. Only one patient has lost his BM-CR after being off therapy for 24 months, he has also lost his CHR and best MR (#13, Fig. 6).

At present, therefore, 5 of 6 evaluable patients are in BM-CR after stopping therapy, and are maintaining their BM-CR for a median time of follow-up after therapy discontinuation of 48 months (range 32–68).

## Discussion

We report on histomorphological BM responses in 58 patients with ET/PV treated with PEG-IFN-α-2a in a prospective, single-center, phase 2 clinical trial after a therapy for a median of 84 months. Our current research substantiates existing data showing that long-term therapy with PEG-IFN-α-2a is not only able to induce CHR and CMR, but could also lead to complete normalization of BM morphology in a subset of patients. Moreover, PEG-IFN-α-2a was capable to completely reverse BM fibrosis in up to 22% of patients, which is higher than previously observed by other investigators [23, 26]. BM responses were achieved after median time on therapy of 48 months, confirming the observation that a long treatment duration is necessary to achieve such a response. Our study also reports a few novel observations. In contrast to previously published results [21], we have noticed significant reduction in BM cellularity in patients with PV who achieved CMR. Patients with BM-CR seemed to derive the most benefits from the therapy with longest duration of initial responses (CHR and CMR), higher rates of CMR, and the fewest disease-related complications. Importantly, none of the patients who had achieved BM-CR progressed to overt MF, and majority of patients who had lost their initial BM-CR have maintained their hematologic and molecular responses. BM-CR was durable, as 69% of them were sustained at the time of last follow-up. Furthermore, 5 of 6 (83%) patients who continued to have BM evaluations after treatment discontinuation, have maintained their BM-CR after a median duration off therapy of 48 months. However, 2 of them have lost their best MR at the time of last follow-up, and one patients has lost his hematologic response.

Despite the fact that patients with BM-CR appeared to benefit from the therapy the most, we could not identify any predictive markers of or a correlation between the achievement of hematologic/molecular responses and BM response. Achievement of BM response or its durability was not essential for obtaining hematological or molecular response or clinical benefit (i.e., remain symptom free with no organomegaly or thrombotic event), and therefore can’t be considered as major point for a treatment decision making in a clinical practice.

One would expect that loss of HR or MR will be accompanied by the loss of BM response; and it might happen in these patients over time. However, in spite of the loss of HR and MR, these patients remain symptoms free without vascular complications while off any active therapy. The fact that their BM is not showing signs of MPN, yet their laboratory parameters (hematologic or molecular) indicate the presence of disease, further echoes our limited understanding of the dynamics of these responses, and its clinical significance. Moreover, it also introduces a question about the definition of useful objective and clinically meaningful response in these patients, as simply relying on the hematologic parameters in a patient with no clinical signs of disease may not be accurate, especially after or during therapy with a biological agents such as interferon. Hopefully, with longer follow-up and better “response” assessment tools, e.g. deep genome sequencing, we will gain better insight into this dilemma.

Notwithstanding its unclear clinical meaning, our results show the ability of PEG-IFN-α-2a to reverse BM fibrosis, confirming its significant biological activity. Furthermore, the observation that these responses could be sustained even after treatment discontinuation, reinforces its strong immunomodulatory effects with a potential for future treatment approaches. Combining PEG-IFN-α-2a with other therapies that have been shown to possibly reverse or reduce BM fibrosis in patients with MF, should be explored in clinical trials.

However, due to the fact that PEG-IFN-α-2a is capable of controlling the disease only for a limited time and progression (loss of achieved hematologic and molecular response, worsening of marrow fibrosis and even transformation to overt MF or AML) could still occur on therapy without any known biological correlations or predictive markers, we need to improve our ability to identify patients who would benefit the most from the treatment.

Major limitation of our trial is that despite its prospective character, morphologic evaluation was not predefined as one of the study endpoints, and therefore patients were evaluated retrospectively based on availability of BM samples during therapy, directed in part by them staying on therapy/responding, which could cause subjective bias.

In summary, our data confirmed the ability of PEG-IFN-α-2a to induce normalization of BM morphology in a subgroup of ET and PV patients. Although we have noticed that patients with a BM-CR may benefit from the therapy the most, with the longest disease control, no predictive markers to guide clinical decision were found. The significance of these findings as well as the prognostic value of BM response in patients with PV and ET remains incompletely understood. Our findings further highlight the need for more elaborate approach in assessing biological effects of interferon-alfa in ET and PV, to help us understand the heterogeneity and dynamics of clinical, hematologic, molecular and morphologic responses and their significance during long-term therapy with PEG-IFN-α-2a.

## Conclusions

PEG-IFN-α-2a is capable to induce complete BM responses in a subset of ET and PV patients, but its correlation with durable clinically relevant treatment benefit warrants further investigation.

## Abbreviations

AML: acute myeloid leukemia
BM: bone marrow
BM-CR: bone marrow complete response
BM-PR: bone marrow partial response
BM-NR: bone marrow no response
ET: essential thrombocythemia
HR: hematologic response
IWG-MRT: International Working Group-Myeloproliferative Neoplasms Research and Treatment
MR: molecular response
CMR: molecular response, complete [undetectable JAK2V617F allele burden]
PR: molecular response, partial [50% reduction in JAK2V617F allele burden]
mMR: molecular response, minor [20–49% reduction in JAK2V617F allele burden]
MF: myelofibrosis
MPN: myeloproliferative neoplasms
PEG-IFN-α-2a: pegylated interferon alpha-2a
PV: polycythemia vera
